# High potential of escalating HIV transmission in a low prevalence setting in rural Tanzania

**DOI:** 10.1186/1471-2458-7-103

**Published:** 2007-06-09

**Authors:** Khadija I Yahya-Malima, Mecky I Matee, Bjørg Evjen-Olsen, Knut Fylkesnes

**Affiliations:** 1 Centre for International Health, University of Bergen, Bergen, Norway; 2 School of Nursing, Muhimbili University College of Health Sciences, Dar es Salaam, Tanzania and Ministry of Health (P.H.N., Morogoro), Africa; 3 Department of Microbiology and Immunology, Muhimbili University College of Health Sciences, Dar es Salaam, Tanzania, Africa; 4 Haydom Lutheran Hospital, Mbulu District, Manyara, Tanzania, Africa

## Abstract

**Background:**

Previous surveillance among antenatal clinic (ANC) attendees within the remote rural Manyara and Singida regions in Tanzania identified an imminent but still, relatively low HIV epidemic. We conducted a population-based HIV study to identify risk factors and validate the representativeness of ANC-based estimates.

**Methods:**

Using a two-stage cluster sampling approach, we enrolled and then interviewed and collected saliva samples from 1,698 adults aged 15–49 years between December 2003 and May 2004. We anonymously tested saliva samples for IgG antibodies against HIV using Bionor HIV-1&2 assays ^®^. Risk factors for HIV infection were analysed by multivariate logistic regression using the rural population of the two regions as a standard.

**Results:**

The prevalence of HIV in the general population was 1.8% (95%CI: 1.1–2.4), closely matching the ANC-based estimate (2.0%, 95% CI: 1.3–3.0). The female to male prevalence ratio was 0.8 (95%CI 0.4–1.7). HIV was associated with being a resident in a fishing community, and having recently moved into the area. Multiple sexual partners increased likelihood of HIV infection by 4.2 times (95% CI; 1.2–15.4) for men. In women, use of contraceptives other than condoms was associated with HIV infection (OR 6.5, 95% CI; 1.7–25.5), while most of the population (78%) have never used condoms.

**Conclusion:**

The HIV prevalence from the general population was comparable to that of pregnant women attending antenatal clinics. The revealed patterns of sexual risk behaviours, for example, close to 50% of men having multiple partners and 78% of the population have never used a condom; it is likely that HIV infection will rapidly escalate. Immediate and effective preventive efforts that consider the socio-cultural contexts are necessary to reduce the spread of the infection.

## Background

Few countries have presented evidence of declining trends in HIV incidence [1-8]. However, there are signs of escalating rural epidemics even within countries reporting a decline of HIV prevalence [4,6,8]. Unfortunately relatively little information is available in rural settings, especially in remote areas with difficult terrain where the dynamics and epidemiology of HIV infection is likely to be different.

To estimate HIV prevalence in remote rural Manyara and Singida regions in Tanzania, we set up a local HIV surveillance system in selected antenatal clinics (ANC). The antenatal surveys conducted at the same clinics at different points in time, identified an imminent but still relatively low HIV epidemic within the catchment area [9,10]. As a follow up, we conducted a population-based-survey to validate the representativeness of the ANC-based HIV surveillance, and to examine sexual risk behaviours among men and women in the general population in the same catchment area.

## Methods

### Study area

We conducted this study from December 2003 to May 2004 within the rural areas of Manyara and Singida in Tanzania that fall within the area served by Haydom Lutheran Hospital (HLH). The HLH is a 400-bed hospital owned and run by the Mbulu Diocese of the Evangelical Lutheran Church of Tanzania (ELCT), and provides the main hospital level care. The area constitutes three administrative divisions, two divisions from the Manyara region, namely Dongobesh from Mbulu district and Basotu from Hanang district, and the Nduguti division from Iramba district, Singida region. This study is part of a comprehensive HIV prevention and intervention programme launched by the hospital to combat HIV in the area [11]. Based on the National 2002 census with extrapolation to 2003 of 3.8% and 2.3% annual growth for the Manyara and Singida regions respectively, the three rural divisions had a total population of 259,292 persons with a male/female ratio of 1.03[10,12]

The study area is unique in that it includes the four main language groups of East Africa. The Iraqw (Cushitic), Datoga (Nilotic), and Iramba (Bantu) are the largest groups, with a smaller group of the indigenous Hadzabe (Khoisan) also represented. Although the main spoken language is Kiswahili, many of those who have not had schooling only speak the local languages. The main economic activities in the area are subsistence farming and pastoralism. We defined a "rural" residential area in our study as villages within the catchment area with more than two general utility shops and an established health facility, while "remote rural" villages are those found in the remote difficult terrain, usually lacking health facilities or utility shops.

### Sampling

In the sample size determination, we assumed a population prevalence of 1.9% based on the antenatal survey estimate [10], the acceptable precision to be 0.92–2.88%, a design effect of two, and 95% confidence interval (CI). This gave a designated sample of 1,700 persons.

The sampling frame had to be units of households to estimate the eligible population for the study. We sampled households in villages representing the catchment area of the ANC-based surveillance system [13]. Administratively, a village consists of smaller sub-villages; each sub-village consists of units of households (an average of 12 households per leader) that fall under one administrative leader locally known as "balozi". In the first stage, we used a two-thirds sampling fraction, and randomly selected clusters of balozi leaders from a list of total balozi leaders from each village to acquire a representative sample. For study purposes, we named "household clusters" to represent the sampled the balozi households. Total clusters of 52 "balozi" (range 21–30 per area) and their respective households' clusters were included in the survey.

We included individuals aged between 15–49 years living within the sampled household clusters in the area during the period of survey. To minimise non-response, we conducted three field re-visits at different times and on different days to enrol the maximum number of eligible residents who were not available at the time of the visit. We informed participants about the voluntary participation and their right to refuse to participate, and that the HIV-1 testing would be done anonymously for surveillance purposes. Individuals wishing to know their test results had to consult the Haydom Voluntary AIDS Control Programme (HAVACOP) for voluntary counselling and testing (VCT), care and treatment based on the national guidelines.

### Data collection procedures and testing for IgG anti HIV-1 antibodies in air-dried saliva

The questionnaire used for interview was adapted from previously validated questionnaires used by the Tanzanian HIV/AIDS indicator survey, 2003–2004 [14] and the established population-based HIV surveys in Zambia[2]. To confirm meaning of the questionnaire, we translated the questionnaire into Swahili and re-translated back to English. Information sought included basic demographic details, sexually related behaviours, illnesses, condom utilisation, and the use of contraceptives in women. Saliva samples were collected at the end of the interview from consenting participants using a Mucosafe ^® ^saliva collection device (Bionor, Skien, Norway) according to the manufacturer's instructions. Mucosa ^® ^collection strips were placed between the lower gums and buccal mucosa for 3 to 5 minutes to allow the collection of an adequate sample. Samples were code-marked on site with the same questionnaire code to enable linkage of characteristics of the participants on analysis. Following collection, the absorbent mucosa strip was stored in a sachet at 4°C and transported to the laboratory for analysis within four hours of collection. Screening and confirmatory tests for HIV-1 antibodies in saliva were conducted using Bionor HIV-1 and 2 assays ^® ^(Bionor, Skien, Norway), a rapid enzyme linked immunosorbent assay (ELISA) that has a sensitivity of 100% and a specificity of 98.8% for both serum and saliva testing for anti-HIV antibodies [14-16]. Evaluation of the use of Bionor HIV 1 & 2 assay for testing of HIV antibodies under local conditions resulted in a 98% agreement when dual saliva and serum samples from pregnant antenatal women where tested. After completion of the laboratory analysis, we linked the HIV test results to the full range respondent information.

### Ethical issues and investigations

Both the Norwegian National Committee for Medical Research Ethics and the National Institute for Medical Research (NIMR) in Tanzania gave ethical approval. Respective health and government authorities at regional and district level received copies of the ethical approval, as required for the implementation of the study. At the individual level, we sought an informed verbal consent after detailed explanation of the purpose and benefits of surveillance for participation in the research only. Prospective participants were also informed about the VCT and treatment unit at the hospital and its potential benefits unrelated to refusal or participation in the research.

### Statistical analysis

Data were entered in duplicate using Excel spreadsheet (Microsoft Corporation, Redmond, Washington) and all the statistical analyses were performed using STATA 9.2 (STATA Corporation, College Station, Texas, USA)[17]. We used survey data to assess the population risk profile related to HIV infection in this population. Apart from the general socio-demographic variables, the study included indicators of sexually related risk behaviours. These included condom use at last sex and current number of sexual partners, condom utilisation and associated risk of infection. We examined age at sexual debut at above or below 18 years and a history of sexually transmitted infections as a marker of sexual risk behaviour. To evaluate HIV risk associated with "commercial sex" during the past year in an informal setting, we used a proxy "incentive sex" defined as "receiving or giving any form of payment in exchange for sex".

On multiple logistic regression, variables with p-values < 0.25 on bivariate analysis and assessment of co-linearity were included in the multivariable model. We present odds ratios (OR) with 95% Confidence Intervals (CI). The age sex-specific adjustments were standardised using the standard rural population of the respective regions [12]. To evaluate the effect of HIV test refusals and related bias, we performed sensitivity analysis.

## Results

The total sample included 1,956 adults aged between 15–49 years. The refusal rate for HIV-1 testing was 13% (253/1,956) for those who completed the questionnaire, among whom 46% (117) were men. Seven participants, who refused the questionnaire survey and accepted HIV testing, had no antibodies for HIV, and were not included in the analysis. Five (0.3%) of the 1,703 tested saliva samples gave indeterminate results.

Table 1 shows similar socio-demographic characteristics of both responders and non-responders, who refused HIV testing. Overall, there were only minor differences between these two groups. The only clear difference was that more non-responders than responders reported no income. Table 2 shows HIV prevalence and associated demographic factors. In rural areas, HIV infection was relatively higher among men (3.0%; 1.6–5.0) compared to women (1.3%; 1.3–3.8). We found higher prevalence of HIV infection among men (5.1%; 2.1–10.4) in Basotu compared to men in Mwanga and Muslur village. Women in Basotu had relatively higher HIV prevalence (5.0%; 2.1–10.2) compared to the rest of the villages. Activities related to fishing in Basotu village represent a major source of income unlike the other areas. Higher prevalence of HIV in men was associated with those aged between 35–39 years (4.3%; 1.4–10.1), being married (2.3%; 1.2–4.2), and lacking conventional means of income (1.7%; 1.4–15.5). HIV prevalence tended to be higher (4.6%; 1.5–11.9) in men who had settled within the past year in the locality compared with those who had stayed for more than a year (1.6%; 0.9–3.0). In contrast, in women, higher HIV prevalence was associated with marital separation and being either widowed or divorced (6.7% 1.7–19.3).

**Table 1:** Socio-demographic characteristics between responders and HIV non-responders (15–49 years), 2003–2004

	**Responders**			**Non-responders**		
**Characteristics**	**Total N (%)**	**Men N (%)**	**Women N (%)**	**Total N (%)**	**Men N (%)**	**Women N (%)**
**Total**	**1698**	**764**	**934**	**253**	**116**	**137**
						
**Area**						
Remote	730 (43.0)	361 (47.2)	369 (39.5)	97 (38.3)	45 (38.8)	52 (38.0)
Rural	968 (57.0)	403 (52.8)	565 (59.5)	156 (61.7)	71 (61.2)	85 (62.0)
						
**Village**						
Haydom	385 (22.7)	156 (20.4)	229 (24.5)	43 (17.0)	18 (15.5)	25 (18.3)
Muslur	259 (15.3)	124 (16.2)	135 (14.5)	23 (9.0)	10 (8.6)	13 (9.5)
Basotu	236 (13.9)	117 (15.3)	119 (12.7)	49 (19.4)	27 (23.9)	22 (16.1)
Dangayda	169 (10.0)	83 (10.9)	86 (9.2)	29 (11.5)	16 (13.8)	13 (9.5)
Mwanga	302 (17.8)	154 (20.2)	148 (15.9)	45 (17.8)	19 (16.4)	26 (19.0)
Nduguti	347 (20.3)	130 (17.0)	217 (23.2)	64 (25.3)	26 (22.4)	38 (27.6)
						
**Age group (years)**						
15–19	262 (15.4)	113 (14.8)	149 (16.0)	38 (15.0)	17 (14.7)	21 (15.3)
20–24	363 (21.4)	155 (20.3)	208 (22.3)	58 (22.9)	28 (24.1)	30 (21.9)
25–29	359 (21.1)	149 (19.5)	210 (22.5)	59 (23.3)	26 (22.4)	33 (24.1)
30–34	270 (15.9)	127 (16.6)	143 (15.3)	46 (18.2)	22 (19.0)	24 (17.5)
35–39	198 (11.7)	93 (12.2)	105 (11.2)	24 (9.5)	11 (9.5)	13 (9.5)
40–49	246 (14.7)	127 (16.6)	119 (12.7)	28 (11.1)	12 (10.3)	16 (11.7)
						
**Marital status**						
Single	476 (28.0)	264 (34.6)	212 (22.7)	86 (34.0)	47 (40.5)	39 (28.5)
Married/Engaged/Cohabit	1156 (68.1)	479 (62.7)	677 (72.5)	159 (62.9)	67 (57.8)	92 (67.2)
Separated/Divorced/Widowed	66 (3.9)	21 (2.8)	45 (4.8)	8 (3.2)	2 (1.7)	6 (4.4)
						
**Education**						
None	165 (9.7)	58 (7.6)	107 (11.5)	18 (7.1)	3 (2.6)	15 (11.0)
1 < 7 years	258 (15.2)	105 (13.8)	153 (16.4)	30 (11.9)	13 (11.2)	17 (12.4)
≥7 years	1212 (71.4)	573 (75.0)	639 (68.4)	192 (75.9)	94 (81.0)	98 (71.5)
Student*	63 (3.7)	28 (3.7)	35 (3.8)	13 (5.1)	6 (5.2)	7 (5.1)
						
**Income**						
None	92 (5.4)	57 (7.5)	35 (3.8)	106 (41.9)	52 (44.8)	54 (39.4)
Employed	254 (15.0)	121 (15.8)	133 (14.2)	11 (4.4)	6 (5.2)	5 (3.7)
Farmer/Cattle/Petty	1352 (79.6)	586 (76.7)	766 (82.0)	136 (53.8)	58 (50.0)	78 (56.9)
						
**Duration of stay**						
< 1 year	218 (12.8)	88 (11.5)	129 (13.8)	31 (12.3)	17 (14.7)	14 (10.2)
≥1 year	1480 (87.2)	676 (88.5)	805 (86.2)	222 (87.8)	99 (85.3)	123 (89.8)

* Student currently enrolled in academic institution.

**Table 2:** HIV prevalence in the general population (15–49 years), 2003–2004

**Characteristic**	**Total N (%)**		**HIV % (95% CI)**		**HIV % (95% CI)**
**Overall HIV prevalence**	1698 (85%)		1.8 (1.2–2.6)	**-**	-

		**Men-N (%)**		**Women-N (%)**	

**Crude HIV prevalence**	1698	764	2.0 (1.1–3.3)	934	1.6 (0.9–2.7)
**Adjusted HIV prevalence ^§^**			1.8 (1.8–1.9)		1.5 (1.4–1.5)
					
**Area**					
Remote	730 (43.0)	361 (47.3)	0.8 (0.2–2.2)	369 (39.5)	0.5 (0.1–1.8)
Rural	968 (57.0)	403 (52.8)	3.0 (1.6–5.0)	565 (59.5)	1.3 (1.3–3.8)
					
**Village**					
Haydom	385 (22.7)	156 (20.4)	1.3 (0.2–4.2)	229 (24.5)	1.3 (0.3–3.5)
Muslur	259 (15.3)	124 (16.2)	0.8 (0.0–4.0)	135 (14.5)	0.7 (0.0–4.7)
Basotu	236 (13.9)	117 (15.3)	5.1 (2.1–10.4)	119 (12.7)	5.0 (2.1–10.2)
Dangayda	169 (10.0)	83 (10.9)	1.2 (0.0–5.8)	86 (9.2)	0
Mwanga	302 (17.8)	154 (20.2)	0.7 (0.0–3.2)	148 (15.9)	0.7 (0.0–3.3)
Nduguti	347 (20.4)	130 (17.0)	3.1 (1.0–7.3)	217 (23.2)	1.84 (0.6–4.4)
					
**Age group (years)**					
15–19	262 (15.4)	113 (14.8)	0.9 (0.1–4.3)	149 (20.0)	0.7 (0–4.2)
20–24	363 (21.4)	155 (20.3)	1.3 (0.2–4.2)	208 (22.3)	1.9 (0.6–5.2)
25–29	359 (21.1)	149 (19.5)	2.0 (0.5–5.4)	210 (22.5)	1.4 (0.4–4.5)
30–34	270 (15.9)	127 (16.6)	2.4 (0.6–6.3)	143 (15.3)	2.8 (0.9–7.5)
35–39	198 (11.7)	93 (12.2)	4.3 (1.4–10.1)	105 (11.2)	1.9 (0.3–7.9)
40–49	246 (14.5)	127 (16.6)	1.6 (0.3–5.1)	119 (12.7)	0.8 (0.0–5.3)
					
**Marital status**					
Single	476 (28.0)	264 (34.6)	1.5 (0.5–4.1)	212 (22.7)	2.4 (0.9–5.7)
Married/engaged/cohabit	1156 (68.1)	479 (62.7)	2.3 (1.2–4.2)	677 (72.5)	1.0 (0.5–2.2)
Separated/divorced/widowed	66 (3.9)	21 (2.8)	0	45 (4.8)	6.7 (1.7–19.3)
					
**Education**					
None	165 (9.7)	58 (7.6)	1.7 (0.1–10.5)	107 (11.5)	0.9 (0.1 – 5.9)
<7 years	258 (15.2)	105 (13.7)	1.9 (0.3–7.4)	153 (16.4)	2.0 (0.5 – 6.1)
≥7 years	1212 (71.4)	573 (75.0)	2.1 (1.1–3.7)	639 (68.4)	1.7 (0.9 – 3.2)
Student*	63 (3.7)	28 (3.7)	0	35 (3.8)	0
					
**Income**					
None	92 (5.4)	57 (7.5)	1.7 (1.4–15.5)	35 (3.8)	0
Employed	254 (15.0)	121 (15.8)	5.3 (0.3–6.4)	133 (14.2)	2.3 (0.6–7.0)
Farmer/cattle/petty	1352 (79.6)	586 (76.7)	1.7 (0.9–3.2)	766 (82.0)	1.6 (0.9–2.8)
					
**Duration of stay**					
< 1 year	218 (12.8)	88 (11.5)	4.6 (1.5–11.9)	129 (13.8)	1.6 (0.3–6.1)
≥1 year	1480 (87.2)	676 (88.5)	1.6 (0.9–3.0)	805 (86.2)	1.6 (0.9–2.8)

§Adjusted by standard total rural population in the two regions

* Student currently enrolled in academic institution.

**Figure 1:**
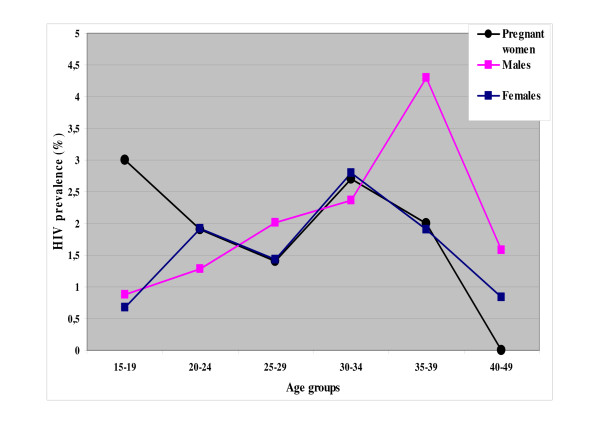
Age- and sex-specific HIV-1 prevalence (%) among antenatal attendees and the general population, 2003–2004.

Figure 1 illustrates the comparisons of HIV prevalence based on results from the ANC-based surveillance and from this population-based survey. Apparently, pregnant and non-pregnant women aged 20–49 years show a similar pattern of infection. However, for the younger age group, women in the general population show a relatively lower HIV prevalence compared to the pregnant women, 0.7% and 3%, respectively. Peak HIV prevalence in women was between the ages of 30 and 34 years, while for men the highest prevalence was between 35 and 39 years. Tables 3 shows the results of the analysis using logistic regression on demographic and selected behavioural indicators adjusted for age. An increase in HIV infection is associated with being a resident in rural areas (OR 4.9; 95% CI 1.2–1.9) and a shorter duration of stay at the present residential address (OR 5.9; 95% CI 1.2–33.3) for men. A four-fold increase of HIV infection was weakly associated with residing in the fishing village of Basotu for both sexes (4.1; 1.0–16.4). In women, the use of contraceptives other than condoms increased the likelihood of HIV infection seven times (OR, 7.7; 95%CI: 2.0–30.2). Men who have received any kind of incentive in exchange for sex, were likely to be infected (OR 6.9; 95% CI 1.2–41.5) compared to those offering the incentive (OR 0.6; 95% CI 0.1–4.0).

**Table 3:** Association between HIV infection and socio-demographic and selected behavioural indicators analysed by univariate analysis

**Men (n = 741)**				**Women (n = 904)**		
**Characteristic**	**N (%)**	**OR (95%CI)**	**P-value**	**N (%)**	**OR (95%CI)**	**P-value**
						
**Residential area**						
Remote	345 (46.6)	**1**	-	369 (39.5)	1	-
Rural	396 (53.4)	**4.9 (1.2–19.9)**	0.03	565 (60.5)	4.6 (0.9–22.9)	0.06
						
**Village**						
Haydom	153 (20.7)	1	-	220 (24.3)	1	-
Muslur	118 (15.9)	0.6 (0.1 – 6.0)	0.61	133 (14.7)	0.6 (0.1–5.0)	0.61
Basotu	117 (15.8)	**4.1 (1.0 – 16.4)**	**0.05**	116 (12.8)	**4.1 (1.1–14.7)**	**0.03**
Dangayda	79 (10.7)	0.9 (0.1 – 6.1)	0.93	81 (9.0)	-	-
Mwanga	148 (20.0)	0.5 (0.1–5.6)	0.58	142 (15.7)	0.5 (0.1–5.4)	0.59
Nduguti	126 (17.0)	2.5 (0.4–15.3)	0.30	212 (23.5)	1.5 (0.4–6.0)	0.58
						
**Age group (years)**						
15–19	90 (12.2)	-	**-**	119 (13.2)	-	-
20–24	155 (20.9)			208 (23.0)		
25–29	149 (20.1)			210 (23.2)		
30–34	127 (17.1)			143 (15.8)		
35–39	93 (12.6)			105 (11.6)		
40–49	127 (17.1)			119 (13.1)		
						
**Marital status**						
Single	241 (32.5)	1	-	183 (20.2)	1	-
Married/engaged/cohabit	479 (64.6)	0.62 (0.1–3.8)	0.61	676 (74.8)	0.2 (0.0–1.3)	0.10
Divorced/separated/widow	21 (2.8)	-	-	45 (5.0)	1.9 (0.2–16.8)	0.55
						
**Duration of stay at current residence**						
> 1 Year	655 (88.4)	1	**-**	777 (86.0)	1	-
≤1 Year	86 (11.6)	**5.9 (1.2–33.3)**	**0.03**	127 (14.0)	1.8 (0.4–7.7)	0.46
						
**Education level**						
None	58 (7.8)	1	-	107 (11.8)	1	-
< 7 years	110 (14.8)	0.6 (0.1–7.5)	0.50	158 (16.9)	3.8 (0.4–4)	0.24
≥7 years	573 (77.3)	0.7 (0.1–5.4)	0.60	639 (70.7)	2.3 (0.3–16.5)	0.40
						
*Income						
None	38 (5.1)	1	-	20 (2.21)	-	-
Employed	94 (12.7)	1.6 (0.1–12.6)	0.63	123 (13.61)	0.3 (0.0–3.7)	0.36
Farmer/pastoralist	609 (86.8)	0.5 (0.1–2.6)	0.38	761 (84.18)	0.8 (0.2–3.5)	0.78
						
**Age at first sex**						
≥18 years	457 (61.7)	1	-	590 (65.27)	1	-
< 18 years	284 (38.3)	2.0 (0.7–6.0)	0.21	314 (34.73)	2.2 (0.8–6.0)	0.12
						
**Ever condom**						
No	541 (73.0)	1	-	737 (81.5)	1	-
Yes	200 (27.0)	0.5 (0.09–2.5)	0.37	167 (18.5)	1.2 (0.3–5.0)	0.83
						
**Multiple sex partner(s)**						
No	393 (53.0)	1	**-**	605 (66.9)	1	-
Yes	348 (47.0)	**5.6 (1.5–20.8)**	**0.01**	299 (33.1)	**4.6 (1.3–16.9)**	**0.04**
						
**History of STIs**						
No	628 (84.8)	1	-	674 (74.8)	1	-
Yes	113 (15.3)	**4.6 (1.4–15.6)**	**0.01**	230 (25.4)	1.6 (0.7–3.9)	0.29
						
**Ever VCT**						
No	512 (69.1)	1	1	567 (62.7)	1	-
Yes	229 (30.9)	1.3 (0.5–3.1)	0.57	337 (37.3)	0.9 (0.4–2.1)	0.77
						
**Uses of contraceptives other than condoms**						
No	-	-	-	576 (63.7)	1	-
Yes				386 (36.3)	**7.7 (2.0–30.2)**	**0.008**
						
**"Incentive sex "**						
**Offer incentive for sex**						
**No**	684 (92.4)	1	-	899 (99.5)	**-**	
**Yes**	56 (7.6)	0.6 (0.1–4.0)	0.55	5 (0.6)		
						
**Receive incentive for sex**						
**No**	731(98.9)	**1**	-	859 (95.0)	1	**-**
**Yes**	8 (1.1)	**6.9 (1.2–41.5)**	**0.03**	45 (5.0)	2.2 (0.5–10.8)	0.33

Adjusted for age.

* None of the women without income had HIV infection; the reference category was thus dropped.

Being a resident in Basotu village (OR 4.0, 95%CI; 1.6–10.3) was an important factor that contributes to an increased risk of HIV transmission for both men and women as shown in table 4. Other factors for men include multiple sexual partners (OR 4.2, 95%CI; 1.2–15.4) and a history of sexually transmitted infections (OR 3.9, 95%CI; 1.1–13.9). In women, the use of contraceptives other than condoms was important for an increased risk of HIV infection (OR 7.7; 95%CI: 1.2–30.2). In the general population, 64% of women did not use any contraceptives. Among users, seven (0.77%) currently sexually active women used condoms.

**Table 4:** Risk factors for HIV infection in adults (15–49 years) on multivariate analysis

**Characteristic**	**Men OR (95%CI)**	**Women OR (95% CI)**
**Residence**		
Other villages	1	1
Basotu	4.0 (1.6–10.3)	4.0 (1.6–10.3)
	**(p = 0.03)**	**(p = 0.02)**
		
**Multiple sex partner(s)**		
No	1	
Yes	4.2 (1.2–15.4)	-
	**(p = 0.02)**	
		
**Duration of stay**		
More than I year	1	
Less than 1 year	5.4 (1.2 – 23.9)	-
	**(p < 0.01)**	
		
**History of STIs**		
No	1	
Yes	3.9 (1.1–13.9)	
	**(p = 0.04)**	-
		
**Contraceptive use other than condoms**		
No		1
Yes	-	6.5 (1.7 – 25.5)
		**(p < 0.01)**
		
**Receive incentive for sex**		
**No**	**1**	-
**Yes**	**6.1 (1.1–35.1)**	
	**(p = 0.04)**	

Among 1,698 participants, 97% (1645) had been sexually active within the past year, and of these, 55% (904) were women. The mean age at first sex was 17 years ± 3 SD. Sexually inactive individuals belonged to the age group 15–19 years. The majority of currently sexually active individuals, 78% (n = 1278), have never used a condom. Among the youngest sexually active age group, 88% had never used condoms. The most important reasons for not using a condom were values based on traditions and religion (91–92%) for both men and women, though a smaller proportion (31% men and women) reported access difficulties associated with cost and availability.

### Sensitivity analysis of adult HIV prevalence based on total non-response

We performed sensitivity analysis (results not shown) under two scenarios. In the first scenario, the relative risk of HIV infection non-responders/responders was 1.5 whereas the relative risk being two in the second scenario. The calculated HIV prevalence in scenarios 1 and 2 would be 2.3%, 95% CI (1.6 – 3.1) and 2.4%, 95% CI (1.8 – 3.3).

## Discussion

An HIV prevalence of 1.8% (95% CI; 1.2–2.6) in the general population aged 15–49 years is relatively low compared with other rural areas in the country [18-20] and elsewhere in Eastern Africa [21]. However, HIV entered these remote communities rather recently, as indicated by the very few cases found as late as the mid-1990s, and moderately increased a decade later [9-11,13]. Furthermore, the revealed patterns of sexual behaviour, particularly the combination of a high proportion with multiple sexual partners, history of sexually transmitted infections and insignificant condom use, are indicative of a great potential of this epidemic to increase in the near future. The higher prevalence of HIV observed in the fishing community underscores the need for focused preventive initiatives that will consider the local social-economic factors that potentially increase HIV transmission.

Population-based surveys are always prone to different types of bias. Firstly, non-response bias, particularly that due to refusal, might be of particular concern in HIV surveys. In this survey, the 13% who refused to participate did not differ significantly from responders by socio-demographic characteristics. Although one cannot exclude the possibility that non-responders differed in prevalence from responders, the low refusal rate and the similarities in socio-demographic characteristics indicate non-response bias may not have significantly biased the results. The close match between the local ANC-based surveillance and the population-based survey from the same areas is suggestive of the representativeness of pregnant women in estimating the local general adult HIV prevalence albeit the inherent biases [2,22,23]. Secondly, at the time of the survey the two regions were experiencing severe drought and food shortage, as well as a lack of natural water sources for cattle. We may have missed some community members (empty or abandoned houses), especially in Dangayda village. This is due to the temporary migration that was necessary in order to search for better pasture for cattle. Though it is likely that non-responders have higher risks of HIV infection compared to responders, we found no difference in socio-demographic factors between them. Thirdly, we lacked a reliable listing of household members and estimated the number of eligible participants within households using household clusters from a list of balozi leaders. This may have led to uncertainty in the estimates of the actual eligible population. Thus, for the reasons above, the uncertainty of the estimated prevalence will always remain.

The use of saliva for HIV antibody testing is particularly well suited for the screening of large populations as well as hard-to-reach populations in non-clinical settings, such as this rural area. A large number of specimens can be collected within limited time and the collection technique does not require specialised training skills. Moreover, studies have shown that testing oral fluids for HIV antibody testing has excellent sensitivity and a specificity for both low and high risk populations [24-26].

Our findings indicate a beginning HIV epidemic in this rural community, as opposed to the general stabilisation of HIV infection in the country as a whole [27]. In early stages of the HIV epidemic, most infections occur in individuals with higher sexual risk behaviours than the general population, and in our study men presented a higher risk of infection [28]. Infection in men occurs at older ages than in women, as has been demonstrated by other studies as well as ours. The male to female ratio of 0.8 puts men in the general population at higher risk, and this suggests that men play a significant role in spreading HIV infection in this area.

The positive association of mobility and increased HIV transmission risks has been shown in other studies [29,30], and in our study it is likely that those who have recently moved into the area are relatively more mobile compared with the permanent residents. The shift of infection from more urban sites is evident, as indicated by the "rural/remote" prevalence ratio of 3.75. This seems to suggest that the "rural HIV epidemic" might similarly increase the remote HIV prevalence, as reported by other studies [31]. The "rural" site in this setting resembles an urban site where different economic activities prevail with the related social mixing patterns that may be scenarios where sexually transmitted infections are common.

We found relatively higher odds of HIV infection in one village where fishing is among the most important sources of income. This village is among the "rural" strata and considered relatively affluent. This observation is in keeping with findings reported in recent studies that have demonstrated increased HIV risk among those involved in fishing [32]. It is likely that fishing provides a steady income compared to farming and cattle keeping, and thus provides a power of influence to engage in commercial sex or to negotiate sex, or alcohol consumption leading to high-risk sexual practices.

Higher risks in men is associated with multiple sexual partners, either by polygamous marriages, infidelity or multiple sexual relationships that are culturally acknowledged [33]. Higher prevalence in men poses a potential threat to women in this area, due to the higher vulnerability of women created by socio-cultural values that deny them the power of negotiation in sexual relationships with their partners [34]. Among selected behavioural indicators, the "mean age at first sex" (from 17 years or more) is no different from the national estimate [13]. Since the majority of the population (62%) begin their first sexual experience at ages above 18 years, 38% of young people have an increased risk of HIV transmission by their early age of sexual experience. More importantly, the low condom utilisation by the younger age group further magnifies their exposure to HIV transmission risk since a very low proportion of this group (6.2%) used condoms at last sexual activity.

The predominant non-utilisation of condoms possibly based on religious and traditional values, irrespective of associated sexual risks, places this community at a higher risk of HIV and other sexually transmitted diseases. The results of the proxy measure of commercial sex "incentive sex" suggests that men who receive gifts of any kind for sex, are more likely to be infected compared to those who offer the incentive. We are uncertain of the existence of commercial sex by men, and who would be the recipients of such transactions since the main mode of HIV transmission has predominantly been heterosexual in most of sub-Saharan African countries. Studies on HIV transmission between men are scarce, and almost non-existent in Africa [35]. An anthropological study from the same area identified fertility as a central means of achieving status in this community [33]. Thus, measures such as condoms may be in conflict with this dominant socio-cultural norm. Under such circumstances, an increase in condom utilisation is unlikely within a short time. Similarly, a recent critique on international indicators of sexual risk behaviour elaborated the variability of interpretation of these indicators to assess sexual risk for HIV infection mainly due to the different factors that occur in various communities [36]. We are of the opinion that local understanding of sexual related behaviours based on socio-cultural contexts in this setting and community participation would have important consequences for designing and evaluating efforts to encourage self-protective behaviours. Based on these factors and our findings, we are likely to observe an escalation in the HIV prevalence in this setting. A similar situation was reflected by an established cohort within rural Mwanza in Northern Tanzania where an increasing HIV incidence/prevalence was observed despite various interventions in the community [37]. Changes in sexual behaviour had not been observed in the latter setting, reflected in findings from the rest of the country, highlighting the difficulty in achieving behavioural changes [13,38].

Sexual risk behaviours revealed in this survey, such as multiple sexual partners and mobility, are well known risk factors for HIV transmission that are not different form elsewhere in Africa, [39,40]. Despite these factors, the HIV prevalence has remained relatively low in this area when compared to other areas within Tanzania and elsewhere in Eastern Africa. Thus, identifying factors involved in maintaining the relatively low HIV prevalence in this setting to-date will be a key point in controlling the spread of HIV. There is convincing evidence that male circumcision has a protective effect against HIV transmission [41-44]. This factor is likely to have significantly contributed since male circumcision is a norm in this area. We can also hypothesize that the initial remoteness, lack of infrastructure, and hence, limited mobility, may have been protective. However, there are ongoing improvements in roads and trade leading to increasing mobility. These developments and the fact that HIV has infected close to 2% of the adult population aged 15–49 years need urgent and effective responses in terms of efforts to reduce transmission risk in this area. In this regard, there are particular challenges in reducing cultural practices that are likely to magnify the recently introduced virus. For instance in some tribes, the "rata" practice that essentially allows women to have "sexual and procreative relationships with a husband's clan brothers (rata)" [34]. Furthermore, there are obvious difficulties in identifying high-risk core group such as sex workers in a setting where sex work is informal.

## Conclusion

In summary, the HIV prevalence from the general population was comparable to that of pregnant women attending antenatal clinics. We have identified a young and apparently potentially increasing HIV epidemic. The revealed patterns of sexual risk behaviours for example close to 50% of men having multiple partners and 78% of the population have never used a condom; it is likely that HIV infection will rapidly escalate. Immediate and effective preventive efforts that take into account the socio-cultural contexts are necessary to reduce the spread of the infection.

## Competing interests

The author(s) declare that they have no competing interests.

## Authors' contributions

All the authors contributed to the paper. All the authors conceived the study. The first author conducted the study. B. Evjen-Olsen and M. I. Matee supervised the research. K. I. Yahya-Malima and K. Fylkesnes led the analysis. All the authors helped to conceptualise ideas and interpret the findings, and reviewed drafts of the manuscript.

## Pre-publication history

The pre-publication history for this paper can be accessed here:


